# FGF9 Promotes Expression of HAS2 in Palatal Elevation via the Wnt/β-Catenin/TCF7L2 Pathway

**DOI:** 10.3390/biom12111639

**Published:** 2022-11-04

**Authors:** Yidan Sun, Xiyu Ying, Ruomei Li, Mengjia Weng, Jiajun Shi, Zhenqi Chen

**Affiliations:** 1 Department of Orthodontics, Shanghai Ninth People's Hospital, Shanghai Jiao Tong University School of Medicine, College of Stomatology, Shanghai Jiao Tong University, National Center for Stomatology, National Clinical Research Center for Oral Diseases, Shanghai Key Laboratory of Stomatology, Shanghai Research Institute of Stomatology, Shanghai 200011, China; 2 Department of Stomatology, Huadong Hospital, Fudan University, Shanghai 200040, China

**Keywords:** FGF9, hyaluronic acid, HAS2, TCF7L2, Wnt/β-catenin pathway

## Abstract

Background: *Fgf9* mutation was found in cleft palate patients. Our previous study indicated that *Fgf9* promotes timely elevation of palate by regulating hyaluronic acid (HA) accumulation at embryonic day 13.5 (E13.5). HA is synthesized by hyaluronic acid synthases (HAS) isoforms 1, 2, or 3. However, how FGF9 regulates HA in palatogenesis is still unclear. Methods: Using Ddx4-Cre mice, we generated the *Fgf9^−^^/^^−^* mouse model (with exon 2 deletion). Immunohistochemistry was used to detect the location and expression of HAS2 in WT and the *Fgf9^−^^/^^−^* palate at E13.5. We also predicted the association between *Fgf9* and *Has2* within the developing palate by performing a bioinformatics analysis. The expression of β-catenin, HAS2, and TCF7L2 were verified by Western blotting after knockout of *Fgf9*. Rescue experiments were performed by ELISA in vitro. Results: *Fgf9^−^^/^^−^* mice exhibited 100% penetrance of the cleft palate. A knockout of *Fgf9* confirmed that HAS2 and TCF7L2 expression was positively correlated with FGF9. TCF7L2 binds to the *Has2* promoter, exhibiting the high specificity predicted by JASPAR. Additionally, increased HA expression by BML-284, TCF-dependent agonist, was blocked in *Fgf9^−^^/^^−^* palate because of the significant decline in TCF7L2 expression. Conclusions: FGF9 promotes HAS2 expression via Wnt/β-catenin/TCF7L2 pathway with TCF7L2 activating transcription of *Has2* in the palate.

## 1. Introduction

Cleft palates occur with an incidence of 0.13 to 2.53‰ worldwide [1]. Defects of the palate can only be fixed surgically and necessitate complex and continual therapies. Modern molecular genetic techniques have been used to enhance the understanding of molecular processes underpinning the cleft palate development. A non-syndromic case of cleft palate with the *Fgf9* T > C mutation was detected in 2007 [2]. FGF9, an autocrine or paracrine growth factor, belongs to a sub-family of fibroblast growth factor family proteins. The mouse *Fgf9* gene maps onto chromosome 14qC3, containing three exons and two introns [3,4]. The mouse and human FGF9 are highly homologous, sharing the same sequence, except for one amino acid [5]. Because palatal development in rodents closely mirror to that in humans, using mouse genetic model with cleft palate can help to study signaling pathways [6]. Colvin found that FGF9 is expressed in the developing craniofacial region and showed its spatiotemporal profile [5]. In addition, cleft palate has been noted in *Fgf9^−/−^* mice with 40% penetrance by different knock-out gene sites [7]. In our pilot study, we generated a *Fgf9*^−/−^ mouse model with 100% penetrance of cleft palate, delayed palatal elevation, and decreased hyaluronic acid accumulation [8].

Palatal development in both mice and humans typically occurs in three stages: palatal expansion and elongation before embryonic day 13.5 (E13.5), elevation and contact before E14.5, and fusion before E15.5 [9]. Palatal elevation is an essential process in palatogenesis led by the intrinsic force produced by HA accumulation [10,11]. HA has unique biophysical properties to bind up to 10 times its own weight with water and to enmesh other extracellular molecules [12,13]. The accumulation and hydration of HA in the palatal shelf are thought to generate a physicochemical force, also known as the turgor force [14]. More recent studies have suggested that HA is not only an internal force for palatal elevation but is also a potential promoter of ECM expansion in palatogenesis [15,16]. Without passing through the Golgi apparatus, HAS on the mesenchymal cell membrane synthesizes HA, which is then released into the ECM [17,18]. In mammals, HAS are encoded by *Has1*–*3* that display diverse expression profiles in embryogenesis [19]. Our previous study has reported that the knockout of *Fgf9* in mice could delay palatal elevation and suppress HA accumulation [8]. However, the underlying molecular mechanism has not been elaborated.

To determine how *Fgf9* regulates HA in palatogenesis, we screened genes of related signaling pathways by the microarray data analysis and verified the results via quantitative PCR and Western blotting analysis. Thereafter we focused on the TCF7L2-activated canonical Wnt signaling due to the correlation between *Fgf9*, *Has2*, and the Wnt pathway. TCF7L2, also known as TCF4, is a transcription factor acting as an essential effector in the Wnt pathway [20]. Our in vitro and in vivo experiments showed that FGF9 affected the expression of HAS2 and TCF7L2; the activation or inhibition of the Wnt pathway affected the expression of HAS2 and HA; the mechanisms of BML-284 and DKK-1 helped to support the mechanism of FGF9 affecting the expression of HAS2; database prediction analysis indicated that TCF7L2 might bind to the *Has2* promoter. Therefore, we postulate that TCF7L2 may bind to the promoter regions of target genes *Has2* and activate the transcription. Our findings reveal a novel mechanism of HAS2 expression in palate regulated by *Fgf9*, suggesting that TCF7L2 might be a promising target to reverse the aberrant palatal elevation of *Fgf9* genic mutation.

## 2. Materials and Methods

### 2.1. Generation of Transgenic Mice

Assays for generating the transgenic *Fgf9^+/^^−^* mice have been described earlier [8]. Mice were raised in a specific pathogen-free (SPF) condition at Shanghai Ninth People’s Hospital in accredited animal facilities. Male and female *Fgf9^+/^^−^* were crossed to produce *Fgf9^−/^^−^* (KO), and Wildtype (WT) embryos. Mice were genotyped by PCR. Details of primers are given in Table 1. DNA fragmentation was detected using PCR and then observed by electrophoresis in 2% agarose gel. A 572-bp band and a 900-bp band indicate WT and KO alleles, respectively. The morning that the vaginal plug was detected was defined as E0.5. Pregnant mice in a critical palatal developmental window were euthanized by CO_2_, and thoracotomy was performed to guarantee death. The embryo-filled uterus was removed, and embryos were obtained under a microscope. Following that, the palatal tissues were further separated.

### 2.2. Micro-CT Analysis, Hematoxylin and Eosin (HE) Staining and Masson’s Trichrome (MT) Staining

Skulls isolated from age-matched E18.5 mice (forming an extensive secondary bony palate) were fixed in 75% ethanol. Then, micro-CT images were completed by a Skyscan 1076 micro-CT (SkyScan, Aartselaar, Belgium) with a spatial resolution of 24 µm. Paraffin-embedded embryos were sliced to a 5 µm thickness. Anterior and posterior sections of palatal shelves were bounded by the maxillary first molar tooth buds. A Hematoxylin and Eosin Staining Kit (Beyotime, Haimen, China) was used for HE staining. For MT staining, Masson’s Trichrome Stain Kit (Solarbio, Beijing, China) was used to stain the palatal sections.

### 2.3. Immunohistochemistry (IHC)

Immunohistochemical analysis was performed using an IHC kit (Solarbio, Beijing, China) following the guidelines of manufacturer. IHC was carried out as we previously described [21]. Briefly, tissue sample slides were incubated with primary antibodies (HAS2, 1:200, DF13702; Affinity) at 4 °C overnight. Finally, hematoxylin was applied in counterstaining. The staining intensity of the samples was quantified using the IHC profiler plugin for ImageJ, which deconvolutes the picture into hematoxylin or DAB and rates the intensity of the staining. Images were first differentiated based on their colors (more purplish or more brownish), and then divided into high positive, positive, low positive, and negative groups according to their intensity values. The total contributing portion of different intensity groups for each sample was calculated as the final intensity.

### 2.4. Quantitative RT-PCR

Total RNA was isolated from palate tissue using an animal tissue RNA extraction kit (DP431, Tiangen Biotech, Beijing, China). The RNA was stored at −80 °C. We performed qRT-PCR as previously described [21]. PrimeScript™ RT Reagent Kit (TaKaRa, Tokyo, Japan) was used to prepare cDNA. The cDNA was stored at −20 °C until use. Three replications were conducted for each cDNA sample. A total reaction mixture of 20 µL contained 5 µL cDNA template, 10 µL SYBR Green PCR Master Mix and, 0.3 µL each primer (10 mM) and DNA-free water to 20 µL. Details of the primers are given in Table 1. PCR was amplified under the following conditions: 95 °C for 5 min, followed by 40 cycles of 95 °C for 10 s and 60 °C for 30 s. The relative mRNA expression levels were calculated with the 2^−ΔΔCt^ method. 

**Table 1:** Primers of studied genes.

Gene Name	Primer Sequence (5′-3′)
*Fgf9*	F:GACAATAATTTCCACCTCC F:CACTGGGCTCTAACTCTTC R:ATTTGCTATGCACGGACAC
*Has1*	F:CCACTAGTGCAATCACAGAAAG R:GTGTGGCTTTGTGTCTCAATAG
*Has2*	F:TGTGAGAGGTTTCTATGTGTCCT R:ACCGTACAGTCCAAATGAGAAGT
*Has3*	F:CCGATCTCATTTCTAAGCAAGC R:CCTCTCCTATTTGTAGCTCTGG
*CTNNB1*	F:CCATGATTCCTTCATATTTGC R:GTAATACGGTTATCCACGCG
*TCF7L2*	F:CCCCTGACTTGAACCCACCC R:CCCTCGTCGTCGGATTTGAT
*GAPDH*	F:GGAGCGAGATCCCTCCAAAAT R:GGCTGTTGTCATACTTCTCATGG

### 2.5. Western Blotting

Palates of embryos was extracted at E13.5 days. Western blotting was carried out as previously described [21]. Proteins were transferred to PVDF membranes (Millipore, Billerica, MA, USA), which were incubated overnight at 4 °C with rabbit anti-FGF9 antibody (1:500, ab206408, MA, Abcam), rabbit anti-TCF7L2/TCF4 antibody (1:1000, 13838-1-AP; Proteintech, Wuhan, China), rabbit anti-β-catenin antibody (1:500, ab223075; Abcam), rabbit anti-HAS2 antibody (1:500, ab290669; Abcam), and rabbit anti-GADPH antibody (1:5000, Proteintech, Wuhan, China). Afterwards, these primary antibodies were detected using HRP-conjugated anti-rabbit IgG antibody (Servicebio, Wuhan, China) for 1 h at room temperature. Finally, the protein-specific signals were detected using a Bio-Rad ChemiDoc™ MP Imaging System.

### 2.6. Quantification of Hyaluronic Acid

Hyaluronic acid content was measured with a hyaluronic acid Quantikine enzyme-linked immunosorbent assay (ELISA) kit (CSB-E08121m; HCUSABIO, Wuhan, China). The supernatants of MEPM cells in the exponential growth phase were collected after centrifugation (20 min, 4 °C, 2000× *g*). Then, supernatants were incubated in individual wells at 37 °C for 30 min. After that, the wells were washed with washing buffer following the manufacturer’s instructions. The wells were further treated with hyaluronic acid-binding protein HRP conjugates and incubated for 30 min. After washing, color was developed with TMB. Optical density was evaluated at 450 nm with a microplate reader. Comparing absorbances of the sample with reagent blanks and hyaluronic acid reference solutions led to the calculation of hyaluronic acid concentrations.

### 2.7. GeneMANIA and Jaspar Database

GeneMANIA (http://www.genemania.org (accessed on 31 August 2022)) is a website providing information on genetic interactions, pathways, co-expression, and colocalization. GeneMANIA was used for the analysis of *Fgf9, TCF7K2* and *Has2* co-expressed genes from many public databases (such as GEO, BioGRID, and IRefIndex). Genes was directly input into the search box of GeneMANIA to obtain the network diagram of co-expressed genes.

Jaspar (https://jaspar.genereg.net (accessed on 20 August 2022)) is an open access database of transcription factor binding profiles. The motif information of TCF7L2 binding site can be searched on the database. Additionally, the promoter sequence obtained through NCBI can be input to obtain the correlation coefficient.

### 2.8. Isolation, Culture, and Treatment of Mouse Embryonic Palatal Mesenchymal(MEPM) Cells

The experimental protocol was designed based on the U.S. Guide for the Care and Use of Laboratory Animals (Eighth Edition, 2011) and was approved by the Animals Committee of Shanghai Ninth People’s Hospital. Mice were euthanized at E13.5. The palatal tissue was separated and washed with phosphate-buffered saline. Then, it was transferred to a Dispase II (2.4 U/mL; Roche Diagnostics)-containing medium to obtain MEPM cells, which were cultured in DMEM/F12 medium (HyClone, Logan, UT, USA) with 15% fetal calf serum (FBS, Gemini Bio, CA, USA) in a 5% CO_2_ atmosphere at 37 °C.

Cells were treated with 0, 5, 10, 20, or 50 µg/mL recombinant human FGF9 (rhFGF9, ab50034; Abcam), 10 ng/mL DKK-1 (StemRD, CA, USA), or 10 ng/mL BML-284 (ab145241; Abcam) (0.1% Dimethyl sulfoxide, DMSO, Sigma-Aldrich, MO, USA) for further analysis.

There are four cell-cultured groups:

Group A: Control group WT or KO MEPM cells were mock treated with 0.1% DMSO for 48 h; Group B: Activated group WT or KO MEPM cells were treated with 10 ng/mL BML-284 for 48 h; Group C: Inhibited group WT or KO MEPM cells were treated with 10 ng/mL DKK-1 for 48 h; Group D: Experimental group WT or KO MEPM cells were treated with 10 ng/mL DKK-1 for 24 h, then replaced with a fresh medium and treated with 20 µg/mL rhFGF9 for 24 h.

### 2.9. Statistical Analysis

All experimental data were analyzed using GraphPad Prism 9.3.1 software (San Diego, CA, USA). All data were displayed as mean ± standard error of the mean ($\bar{x}$ ± SEM). Differences were analyzed by one-way analysis of variance (ANOVA). *p <* 0.05 was considered as statistically significant.

## 3. Results

### 3.1. Fgf9^−/−^ Embryos Exhibited Cleft Palate

**Figure 1:**
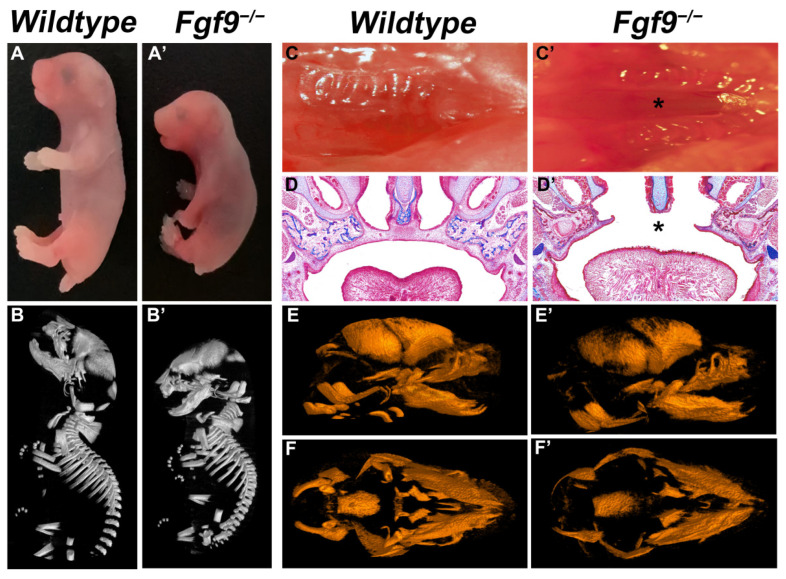
Craniofacial phenotypes of *Wildtype* and *Fgf9^−/^^−^* embryos. (**A**,**B**) Comparison of the size of (**A**) *Wildtype* and (**A’**) *Fgf9^−/^^−^* mice at E18.5 by gross observation (**A**,**A’**) and micro-CT scans (**B**,**B’**). Inferior axial views of (**C**) *Wildtype* and (**C’**) *Fgf9^−/^^−^* E18.5 palates. MT staining of coronal sections in (**D**) *Wildtype*, and (**D’**) *Fgf9^−/^^−^* palates. Lateral views of (**E**) *Wildtype* and (**E’**) *Fgf9^−/^^−^* craniofacial skeletons provided by micro-CT scans. Inferior axial views of (**F**) *Wildtype* and (**F’**) *Fgf9^−/^^−^* craniofacial skeletons provided by micro-CT scans. Black star * indicates cleft secondary palate.

To increase the cleft palate penetrance of mice, we disrupted the expression of *Fgf9* in secondary palate development by mating Ddx4-Cre driver with floxed *Fgf9* (*Fgf9^fl/fl^*) mice. The Ddx4-Cre mouse model is expected to mediate whole-body knockout for palatogenesis studies. After mating male and female *Fgf9^+/−^* mice, the pregnant *Fgf9^+/−^* mice were dissected to obtain progeny *Fgf9^−^^/^^−^* (*KO*) embryos that died within hours of birth with a smaller body size compared with *wildtype* embryos (Figure 1A–B’) and with cleft palate (100% penetrance, *n* = 25, Figure 1C,C’). At E18.5, *KO* embryos displayed morphologically abnormal unfused palatal scaffolds (black star, Figure 1D’). In WT embryos, trabecular bone was complete and thick (Figure 1D) compared to *KO* embryos (Figure 1D’). Micro-CT scanning covered the craniofacial skeleton and further exhibited the developmental abnormalities of the calvarial bones (Figure 1E–F’).

### 3.2. Abnormal HA Accumulation and HAS2 Expression in the Fgf9^−/−^ Embryos

**Figure 2:**
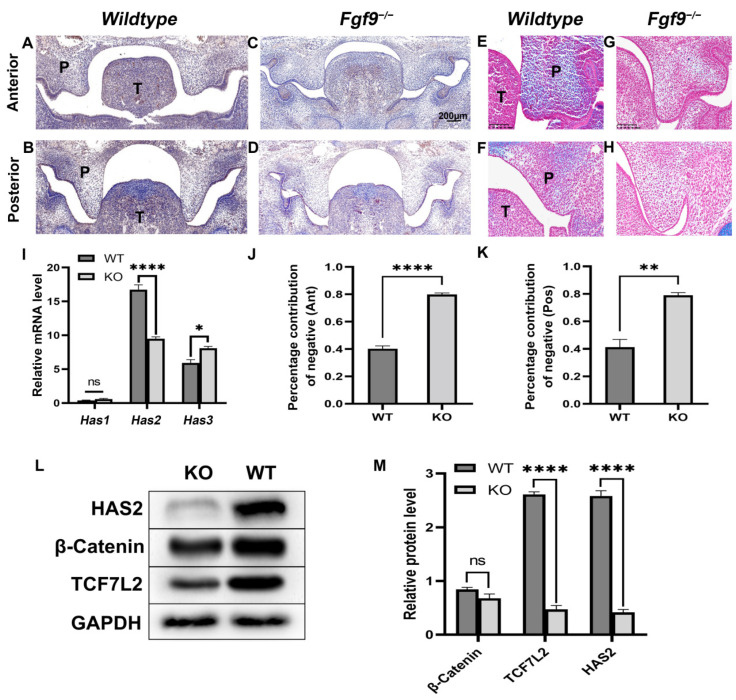
Analysis of HA and HAS2 expression in *Wildtype* and *Fgf9^−/−^* palate at E13.5. (**A**–**D**) Expression of HAS2 in (**A**,**C**) anterior, and (**B**,**D**) posterior coronal sections of *Wildtype* (**A**,**B**) and *Fgf9^−/−^* mutant (**C**,**D**) embryos. (**E**–**H**) Alcian blue-stained (**E**,**G**) anterior, and (**F**,**H**) posterior coronal sections of *Wildtype* (**F**,**F**) and *Fgf9^−/−^* mutant (**G**,**H**) embryos. (**I**) The impact of the knockout *Fgf9* on the mRNA level of *Has1-3* was examined by a qRT-PCR experiment in vivo. (**J**,**K**) *Wildtype* and *Fgf9^−/−^* (**J**) anterior, and (**K**) posterior palatal HAS2 expression was determined by quantification, and the proportion of IHC-negative cell was demonstrated. (**L**,**M**) Western blot analysis showed differences of HAS2, β-catenin, and TCF7L2 expression in *Wildtype* and *Fgf9^−/−^* palate. The bar graph represents the mean ± SEM. ns: no significance, *p* ≥ 0.05, * *p* < 0.05, ** *p* < 0.01, **** *p* < 0.0001. P, palate. T, tongue.

To understand the relationship between HA and *Fgf9^−/^^−^* cleft palate, we examined HA accumulation and HAS2 expression in the *Fgf9^−/^^−^* embryos at E13.5, the critical period for palatal development. We determined the concentration of HA with Alcian blue staining. Palatal ECM including HA is stained in blue, while the color red represents cell nuclei. HA expression was decreased in *Fgf9^−/^^−^* palates compared to that in WT palate (Figure 2E–H). As HA is synthesized by HAS1-3, we then evaluated the WT and *Fgf9^−/^^−^* mice palatal *Has1-3* mRNA expression at E13.5. Our results also showed that, compared to WT mice, *Has2* mRNA expression was significantly decreased in *Fgf9^−/^^−^* mice palate (*p* < 0.05) while *Has1* and *Has3* mRNA expression slightly increased, which may result from the compensation mechanism due to decreased *Has2* expression (Figure 2I). The HAS2 protein expression at E13.5 was consistent with the results of *Has2* gene expression in *Fgf9^−/^^−^* mice which was largely down-regulated compared with the WT mice (Figure 2L,M). Similarly, while HAS2 was prominently expressed in ventral nasal tissues of posterior and the whole anterior palatal regions at the critical points of palatal elevation in WT mice (Figure 2A,B), IHC negative cells were significantly increased in the anterior and posterior *Fgf9^−/^^−^* palates (Figure 2J,K). Our findings demonstrated that HAS2 expression was uniformly decreased in the *Fgf9^−/^^−^* mice palate.

### 3.3. FGF9 Promotes HAS2 and Tcf7L2 Expression in Palate

**Figure 3:**
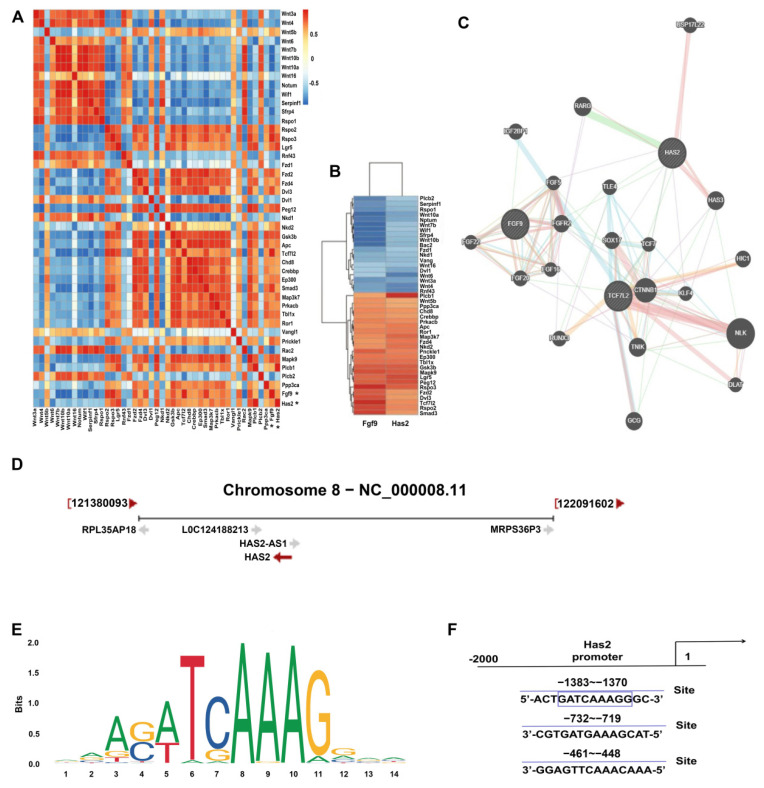
The correlation among FGF9, HAS2 and TCF7L2 in palate development. (**A**,**B**) Cluster thermography analysis of differential expression of *Fgf9, Has2* and WNT/β-catenin pathway during palatogenesis. Correlation was assessed using a Pearson’s correlations test. (**C**) Analysis of FGF9 and HAS2 co-expressed genes through the genemania database. (**D**) Mice *Has2* promoter information was obtained from NCBI. (**E**) The DNA motif for TCF7L2 was obtained from JASPAR. (**F**) TCF7L2 binding sites in *Has2* promoter were predicted by JASPAR.

**Figure 4:**
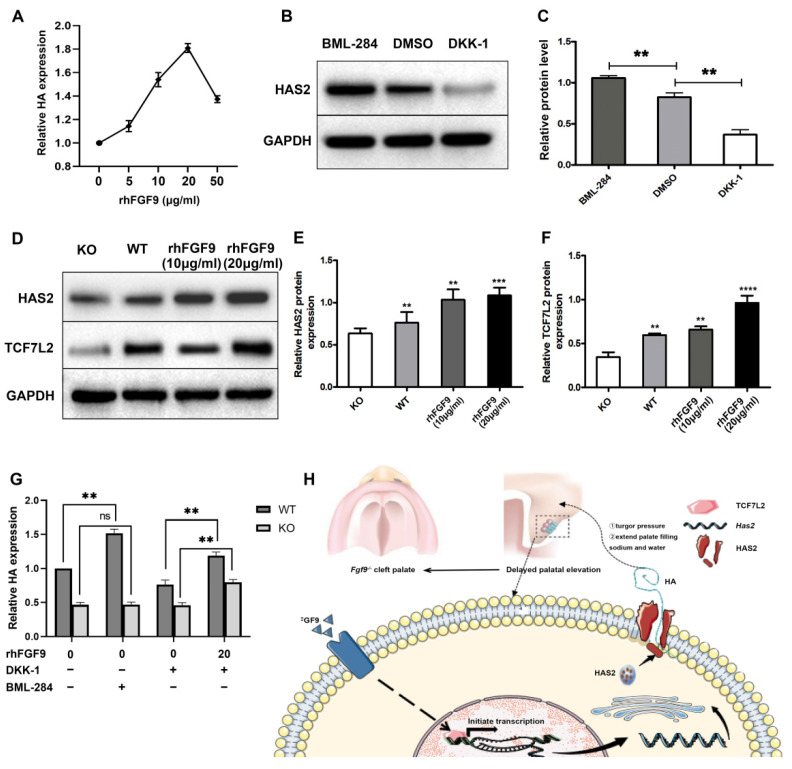
Mechanism of Fg*f9* promoting HA expression. (**A**) Effect of different concentrations of rhFGF9 (0–50 µg/mL) on HA secretion in MEPM. (**B**,**C**) The protein levels of HAS2 in MEPM cells after treatment with BML-284 or DKK-1 were determined by Western blot assay. (**D**–**F**) The protein levels of HAS2 and TCF7L2 in KO MEPM cells and WT MEPM cells treated with 0, 10, or 20 µg/mL rhFGF9 were determined by Western blot assay. Asterisks indicate a significant difference from the KO MEPM cells.(**G**) Elisa analysis of HA expression in WT and KO MEPM with different treatment. (**H**) Schematic diagram of mechanism of this research. The bar graph represents the mean ± SEM. ns: no significance, *p* ≥ 0.05, ** *p* < 0.01, *** *p* < 0.001, **** *p* < 0.0001. Western blot analysis showed differences of HAS2, β-catenin, and TCF7L2 expression in *Wildtype* and *Fgf9^−/−^* palate.

Next, we would like to understand the molecular mechanisms by which FGF9 induced the upregulation of HAS2 in palatal development. The GSE101825 microarray data derived from the GEO database (https://www.ncbi.nlm.nih.gov/geo/ (accessed on 14 October 2021)) were used to analyze the difference in gene expression between Pearson correlations. Analysis of various genes involved in the palate at E14 by treating pregnant mice with cleft palate mice with an agonist. We clustered thermography analysis of differential expression of *Fgf9*, *Has2,* and partial genes of the WNT/β-catenin signaling pathway during palatogenesis. The colors scale is used for representation: when the positive expression correlation is stronger (closer to 1), the red is darker, and when the negative expression correlation is stronger (closer to −1), the blue is darker (Figure 3A). Then, cluster molecules with high correlation coefficients (coefficient >0.5 or <−0.5) for *Fgf9* and *Has2* according to positive or negative expressions (Figure 3B) were analyzed. FGF9 and HAS2 were co-expressed in the Genemania database (Figure 4C). TCF7L2, a transcription factor for the Wnt pathway, interacted with β-catenin to promote its target transcription. There was a decrease in TCF7L2 expression in the palate of *Fgf9^−/^^−^* mice compared to the WT mice. However, β-catenin expression indicated no significant change. Western blot analysis of TCF7L2 and β-catenin was almost in concordance with the mRNA expression data (Figure 2L,M). To further explore the *Has2* promoter core region and transcription factor TCF7L2 binding sites, the mRNA and promoter sequences of *Has2* in the NCBI database were used as the query (Figure 3D). Then, we obtained the DNA motif and identified several TCF7L2-specific binding sites in the *Has2* promoter using the Jaspar database, and we found that the docking scores were larger than 5 (80% threshold), which showed that they possessed good binding activity (Table 2, Figure 3E,F). Thus, big data analytics revealed a correlation between *Fgf9*, *Has2*, and the Wnt pathway. Specifically, FGF9 promotes *Has2* transcription via the TCF7L2-activated Wnt pathway, which in turn facilitates HA accumulation in the palate.

#### Fgf9 Promotes HAS2 Expression via the Tcf7L2-Activated Wnt Pathway In Vitro

Furthermore, we used an in vitro MEPM culture model to determine the effect of FGF9 on HA expression, MEPM cells were treated with various doses of rhFGF9 (0, 5, 10, 20, and 50 µg/mL), respectively, at 48 h. Results indicated HA levels were upregulated with increased FGF9 concentrations, and that HA relative expression was the highest when treated with 20 µg/mL of rhFGF9 (Figure 4A). Results indicated HA levels to be upregulated with increased FGF9 concentrations and HA relative expression was the highest when treated with 20 µg/mL of rhFGF9. Based on the above findings, we used 20 µg/mL of FGF9 for subsequent experiments. To detect the effects of the Wnt pathway on HAS2 protein, relative HAS2 protein expression levels in the control cells, DKK-1 inhibitor and BML-284 activator groups were detected by Western blotting (Figure 4B,C). Control cells were mock treated with 0.1% DMSO. This revealed that activating the Wnt signaling pathway upregulates HAS2 expression in MEPM cells, while decreased expression of the HAS2 protein was detected following inhibition of the Wnt signaling. Then, to detect the effects of FGF9 on HAS2 and TCF7L2 protein, MEPM cells were treated with various doses of rhFGF9 (0, 10, and 20 µg/mL). Results indicated that HAS2 and TCF7L2 levels were upregulated with increased FGF9 concentrations (Figure 4D–F).

To further verify that FGF9 promotes the expression of HAS2 via the Wnt/β-catenin/TCF7L2 signaling pathway, the activator BML-284 (able to enhance TCF-dependent transcriptional activity) and inhibitor DKK-1 (able to bind to the LRP receptors) of the Wnt pathway were used to treat WT and KO MEPM cells at E13.5. KO MEPM cells presented significant reduced HA secretion (Figure 4G). For the inhibited group, DKK-1 added at 48 h resulted in a significant decrease in HA secretion. However, treatment with rhFGF9 after blocking the Wnt/β-catenin pathway with DKK-1 in WT MEPM cells could still increase HA expression. For the activated group, BML-284 added at 48 h resulted in a significant rise in HA secretion in WT MEPM cells, while for the KO MEPM cells, the rising expression of HA was not detected due to the mechanism of activation of its TCF-dependent character. This finding is consistent with our prediction about *Fgf9* regulatory function. *Fgf9* induces *Has2* by activating the WNT/β-catenin/Tcf7L2 pathway in the palate at E13.5, which in turn promotes HA expression to facilitate normal palatal development (Figure 4H).

## 4. Discussion

*Fgf9* has well-established associations with SOX family, FGF family, TGF family, and WNT family members during palatogenesis [22]. Thus, the *Fgf9^−^^/^^−^* embryo is a great model for investigating the genetic interactions involved in palate development.

In recent years, multiple studies have reported the association of weakened HA accumulation with failure of palatal elevation in animal models [9,23,24,25], indicating the intrinsic role for HA in the timely expansion of the palate. HA, the downstream target of *Fgfs* [26,27,28], has special physicochemical features that allow it to bind with huge amounts of water. The hydration of HA is able to produce the turgor force necessary to promote palatal elevation [29]. On the contrary, abnormal palatal expansion prevents the two shelves from contacting after elevation on E14.5–E15.5, resulting in the formation of cleft palate [9,11,17,30]. As assessed by hyaluronic acid binding protein (HAbp) staining [30], the cross-sectional area of the palatal shelf in *Fgf9^−^^/^^−^* embryos had a significant reduction, while nuclear density of mesenchyme cells was significantly increased, at E13.5. This implied that HA plays a crucial role in the expansion of palatal shelf and subsequent palatal elevation. Our results are consistent with the earlier observation that *Fgf9* controls embryonic lung size via regulating mesenchymal expansion [7]. Collectively, we have demonstrated that HA accumulation plays unique role in palatal growth.

*HAS1-3* are the enzymes responsible for HA synthesis and secretion at the plasma membrane. HAS2 predominantly produces large molecular weight of HA (over 2 × 10^6^ Da) [31]. Larger molecular weight of HA is more prone to hydration [32]. Conditional knockout of *Has2* led to cleft palate and failure in palatal elevation, similar to the results of our mouse model [17]. Our results showed that *Has2* gene and protein expressions in *Fgf9^−^^/^^−^* were both largely down-regulated compared with the wild type. On the other hand, *Has1* and, *Has3* expression are slightly increased, which can be explained as being a compensatory mechanism of the down-regulation in *Has2*. Together, we demonstrated that *Fgf9*-induced hyaluronic acid accumulation, synthesized by HAS2, is essential for palatal elevation.

At present, patients with cleft palate still need to go through complex and multidisciplinary treatment. Hence, a better understanding of how molecular interactions drive palatal growth and fusion is critical for preventive strategies or early interventions for cleft palate treatment in humans. As the mechanism of FGF9 regulating HAS2 remained unclear, we carried out a correlation analysis using microarray data extracted from the GEO database [33]. We found a strong correlation between *Fgf9*, *Has2,* and the Wnt/β-catenin pathway. During craniofacial development, the Wnt signaling pathway plays a critical role in regulating human and murine palatal morphogenesis [34,35]. Wnt ligand interacts with its receptor and is frizzled to initiate downstream signaling transduction [36]. LRP6 is a signal receptor protein of the Wnt signaling [37]. Small molecule agonists and antagonists are useful tools for studying embryogenesis in animal models [38]. DKK1, a secreted antagonist of LRP6, can inhibit the Wnt signaling pathway [39]. DKK1 binds with high affinity to LRP6 and induces its endocytosis, thus effectively removing the LRP6 protein from the cell surface and making cells less able to respond to the Wnt ligand [40]. DKK-1 significantly down-regulated HA expression in MEPM cells. Our previous study suggested that DKK-1 limited the level of HA expression. However, when it underwent simultaneous addition treatment of both rhFGF9 and DKK-1, HA expression showed some recovery. When rhFGF9 was added to WT cells treated with DKK-1, the expression of HA was increased. The potential reason might be that rhFGF9 directly acted on TCF7L2 transcription factor and counteracted the inhibition of DKK-1 on the Wnt pathway.

The interaction of the Wnt receptor and ligand leads to the accumulation of β-catenin in the cytoplasm [41]. After that, β-catenin transfers to the nucleus where it combines with TCF to create a heterodimeric complex that controls the expression of the target genes [42]. Firstly, we detected the expression of β-catenin because it is an important effector molecule of this pathway, and indeed most of the target genes in the Wnt pathway regulate the expression of β-catenin protein. Additionally, then, the other important target genes of the Wnt pathway have been screened, but there did not exist a clear molecular relationship before β-catenin exerted effects. So, we focused on the transcription process of the pathway. TCF7L2 protein was significantly decreased in *Fgf9^−^^/^^−^* palate, while the expression of β-catenin protein increased, which means *Fgf9* may be the upstream of Wnt pathway and regulates its signal transduction. TCF7L2 is a known transcriptional effector of the Wnt pathway [43]. The exon–intron organizations of human and mouse TCF7L2 are highly conserved. Knocking down TCF7L2 results in the aberrant palatal contact and a series of abnormal characteristics of mice facial development [44,45]. BML-284 is a TCF-dependent activator of canonical Wnt signaling, enhancing the transcription activity [46,47]. TCF-dependent reporter gene expression induced by BML-284 will be blocked in the presence of the dominant negative TCF7L2. In other words, the presence of TCF is required for the effects of activation of BML-284 [48]. BML-284 significantly upregulated HA expression in WT MEPM cells, whereas, this increased expression was blocked in KO MEPM cells. HA expression in KO MEPM cells should have increased if FGF9 did not influence transcription factor. However, the expression of HA through other ways was inhibited because expression of HA is promoted by BML-284 just like the effect on WT MEPM cells, which indicates that β-catenin and TCF-dependent *Has2* expression induced by BML-284 was interrupted when TCF7L2 was underexpressed.

Our data suggest that during palate development, FGF9 might regulate the expression of HAS2 by controlling the transcription of the Wnt signaling pathway. One limitation of these methods however is that, without a direct intermolecular interaction, it leads to a weakening of the straightforward interpretation. How FGF9 regulates the expression of TCF7L2, and if TCF7L2 binds with the *Has2* promoter directly, remain to be investigated. However, this article provides new insights into the pathogenesis of *Fgf9^−/^^−^* cleft palate, and TCF7l2 might be a promising therapeutic target for early intervention to reverse the aberrant palatal elevation caused by *Fgf9* genic mutation.

## Figures and Tables

**Table 2:** TCF7L2 binding sites in *Has2* promoter were predicted by JASPAR. The closer the relative score is to 1 indicating a tighter binding.

Name	Score	Relative Score	Sequence ID	Start	End	Strand	Predicted Sequence
TCF7L2	13.432302	0.92807441	NC_000008.11	1370	1383	−	ACTGATCAAAGGGC
TCF7L2	9.703565	0.87654208	NC_000008.11	719	732	+	CGTGATGAAAGCAT
TCF7L2	9.428534	0.87274107	NC_000008.11	448	461	+	GGAGTTCAAACAAA

## Data Availability

All data presented in the study are included; further inquiries can be directed to the corresponding author.
